# Atlas-based auto-segmentation for postoperative radiotherapy planning in endometrial and cervical cancers

**DOI:** 10.1186/s13014-020-01562-y

**Published:** 2020-05-13

**Authors:** Nalee Kim, Jee Suk Chang, Yong Bae Kim, Jin Sung Kim

**Affiliations:** 1 Department of Radiation Oncology, Yonsei Cancer Center, Yonsei University College of Medicine, 50-1 Yonsei-ro, Seodaemun-gu, Seoul, 03722 South Korea; 2 Department of Radiation Oncology, Samsung Medical Center, Sungkyunkwan University School of Medicine, 81 Irwon-ro, Gangnam-gu, Seoul, 06351 Korea

**Keywords:** Auto segmentation, Radiotherapy, Gynecologic cancer, Intensity-modulated radiation therapy, Computer-assisted radiotherapy planning

## Abstract

**Background:**

Since intensity-modulated radiation therapy (IMRT) has become popular for the treatment of gynecologic cancers, the contouring process has become more critical. This study evaluated the feasibility of atlas-based auto-segmentation (ABAS) for contouring in patients with endometrial and cervical cancers.

**Methods:**

A total of 75 sets of planning CT images from 75 patients were collected. Contours for the pelvic nodal clinical target volume (CTV), femur, and bladder were carefully generated by two skilled radiation oncologists. Of 75 patients, 60 were randomly registered in three different atlas libraries for ABAS in groups of 20, 40, or 60. ABAS was conducted in 15 patients, followed by manual correction (ABAS_c_). The time required to generate all contours was recorded, and the accuracy of segmentation was assessed using Dice’s coefficient (DC) and the Hausdorff distance (HD) and compared to those of manually delineated contours.

**Results:**

For ABAS-CTV, the best results were achieved with groups of 60 patients (DC, 0.79; HD, 19.7 mm) and the worst results with groups of 20 patients (DC, 0.75; *p* = 0.012; HD, 21.3 mm; *p* = 0.002). ABAS_c_-CTV performed better than ABAS-CTV in terms of both HD and DC (ABAS_c_ [*n* = 60]; DC, 0.84; HD, 15.6 mm; all *p* < 0.017). ABAS required an average of 45.1 s, whereas ABAS_c_ required 191.1 s; both methods required less time than the manual methods (*p* < 0.001). Both ABAS-Femur and simultaneous ABAS-Bilateral-femurs showed satisfactory performance, regardless of the atlas library used (DC > 0.9 and HD ≤10.0 mm), with significant time reduction compared to that needed for manual delineation (*p* < 0.001). However, ABAS-Bladder did not prove to be feasible, with inferior results regardless of library size (DC < 0.6 and HD > 40 mm). Furthermore, ABAS_c_-Bladder required a longer processing time than manual contouring to achieve the same accuracy.

**Conclusions:**

ABAS could help physicians to delineate the CTV and organs-at-risk (e.g., femurs) in IMRT planning considering its consistency, efficacy, and accuracy.

## Background

Intensity-modulated radiation therapy (IMRT) has demonstrated significantly reduced gastrointestinal and urinary toxicity [1, 2]; therefore, it has become popular in postoperative radiotherapy (RT) for gynecologic cancers. Because IMRT enables the delivery of high-precision therapeutic doses to tumors while sparing organs-at-risk (OAR), accurate segmentation of the target volume and OAR is an essential and critical step for intricate RT plans. Despite consensus guidelines for target volume and OAR segmentation [3, 4], inter- and intra-observer variations remain [5, 6]. In general, contours are delineated manually by the radiation oncologist or dosimetrist, and this step requires the majority of time in the entire RT planning process. To overcome this issue, auto-segmentation within the planning process has become crucial.

Various algorithms for auto-segmentation predominantly use deformable image registration for contour generation, which involves the transformation between two images in which the voxels of the moving image sets are skewed to match the voxels of the target image set and during which a deformation vector field is created [7–9]. In atlas-based auto-segmentation (ABAS), segmented structures from atlas libraries are propagated onto a subject image using deformable image registration algorithm. Because multiple-ABAS uses a voting scheme for determining whether a voxel is inside or outside the structure, it is more susceptible to topological artifacts compared with single-ABAS [10]. However, multiple-ABAS could overcome the issues encountered with single-ABAS, such as large discrepancies in volume and location between the atlas library and subject data [11].

Although there are several reports of using ABAS in pelvic RT, especially for prostate cancer [12–18], limited information is available on the impact of library size and change in accuracy after manual revision. Generation of an individualized ABAS library instead of a built-in library is crucial for further clinical implementation in each center. Herein, we aimed to evaluate the accuracy and efficacy of an ABAS algorithm for target volumes and OAR (e.g., bladder and femur) in patients with gynecologic cancer and to evaluate whether ABAS performance could be improved with increasing numbers of patients in each atlas library. In addition, we also evaluated the clinical implementation of ABAS processes.

## Methods

### Patient selection

This study was approved by the Health Institutional Review Boards of Yonsei University Hospital (No. 4–2019-0937). The inclusion criteria were as follows: (1) patients diagnosed with endometrial or cervical cancer after total hysterectomy with negative surgical margins, (2) patients who underwent pelvic CT for postoperative RT, (3) planning CT of 3 mm slice thickness with intravenous contrast, and (4) patients who followed an institutional 2-h bladder filling protocol [19]. Patients who either had remnant uterus or adnexae, had spine or femur deformities (history of surgery), underwent planning CT in a prone position, or underwent partial or total cystectomy were excluded. Overall, 75 patients were randomly selected. Of these 75 patients, 60 were randomly registered to 3 different atlas libraries in groups of 20, 40, or 60. As we only included patients who completed treatment, contours generated by ABAS were never used for treatment planning.

### Manual segmentation

Contours for the whole pelvic nodal clinical target volume (CTV) (RTOG guidelines) [4] and OAR (femur and bladder) [3] were delineated by a single experienced clinician (YB Kim). Due to the considerable variation in the vaginal cuff volume in each patient [20, 21], we excluded vaginal cuff CTV in this study and only evaluated the pelvic nodal CTV. For OAR segmentation, we selected the femurs and bladder to evaluate the feasibility of ABAS for bone and soft tissue structures with different Hounsfield units. Both femurs were delineated separately to investigate the different types of ABAS (i.e., ABAS-femur and simultaneous ABAS-Bilateral-femurs).

### Atlas-based auto-segmentation (ABAS)

The process of ABAS was conducted using the commercial “Atlas Segmentation” in MIM Maestro 6.7 (MIM Software Inc., Cleveland, OH, US). Fifteen sets of CT were used as a test set to evaluate the accuracy and efficacy of ABAS for each library (*n* = 20, 40, and 60). It should be noted that as the n increased for each atlas library (e.g., from an atlas library of *n* = 20 to *n* = 40), the previous group’s atlas elements were retained and those of an additional 20 patients were included to generate a new atlas library (Additional file 1). Detailed information on baseline characteristics of atlas library and test set are summarized in Additional file 2**.**

As the first step in library construction, a template subject was assigned; then, the remaining subjects were registered to the template subject separately. For minimizing the bias and maintaining the consistency of registration alignment, an additional intervention during registration was prohibited. The ABAS algorithm automatically matched the atlas subject in accordance with the input test set. Based on the intensity and a freeform cubic spline interpolation [22], contours of CTV and OARs were deformed, registered, and transferred to the test set.

### Running time

Both ABAS and manually corrected ABAS (ABAS_c_) were performed on a single workstation (Intel® Core™ i7–4770 central processing unit of 3.4 GHz, 32 GB of random-access memory, HP Inc., Spring, TX; Microsoft® Windows® 7 Professional K, Microsoft, Redmond, WA, US). The computation time for ABAS and the manual correction time were recorded.

### Validation method

Contours generated by ABAS and ABAS_c_ were compared with conventional manual contours (M-CTV, M-femur, and M-bladder). For accuracy analysis, both Dice’s coefficient (DC) [23] and the Hausdorff distance (HD) [24] were used. Results of DC were between 0 and 1, where 0 represented no intersection and 1 reflected a perfect overlap of structures. The accepted limit for contours > 30 ml was DC > 0.85 [25]. However, the value of DC was limited owing to local discrepancies [26]. In contrast, HD considered the degree of mismatch between two surfaces based on contour boundaries, eliminating the ambiguity of the volume-based DC metric.

### Statistical analysis

Paired *t*-tests were used to compare the DC, HD, and time values for ABAS and ABAS_c_. Due to the simultaneous nature of the comparisons, a Bonferroni correction was adopted to address the multi-comparison issue. Since there were three groups in the current study, an alpha value of 0.05/3 was used: a *p*-value < 0.017 was regarded as a rejection to the null hypothesis and therefore considered statistically significant. Statistical analyses were performed using SPSS version 25.0.0 (IBM Corp., Armonk, NY).

## Results

### Segmentation accuracy

**Fig. 1:**
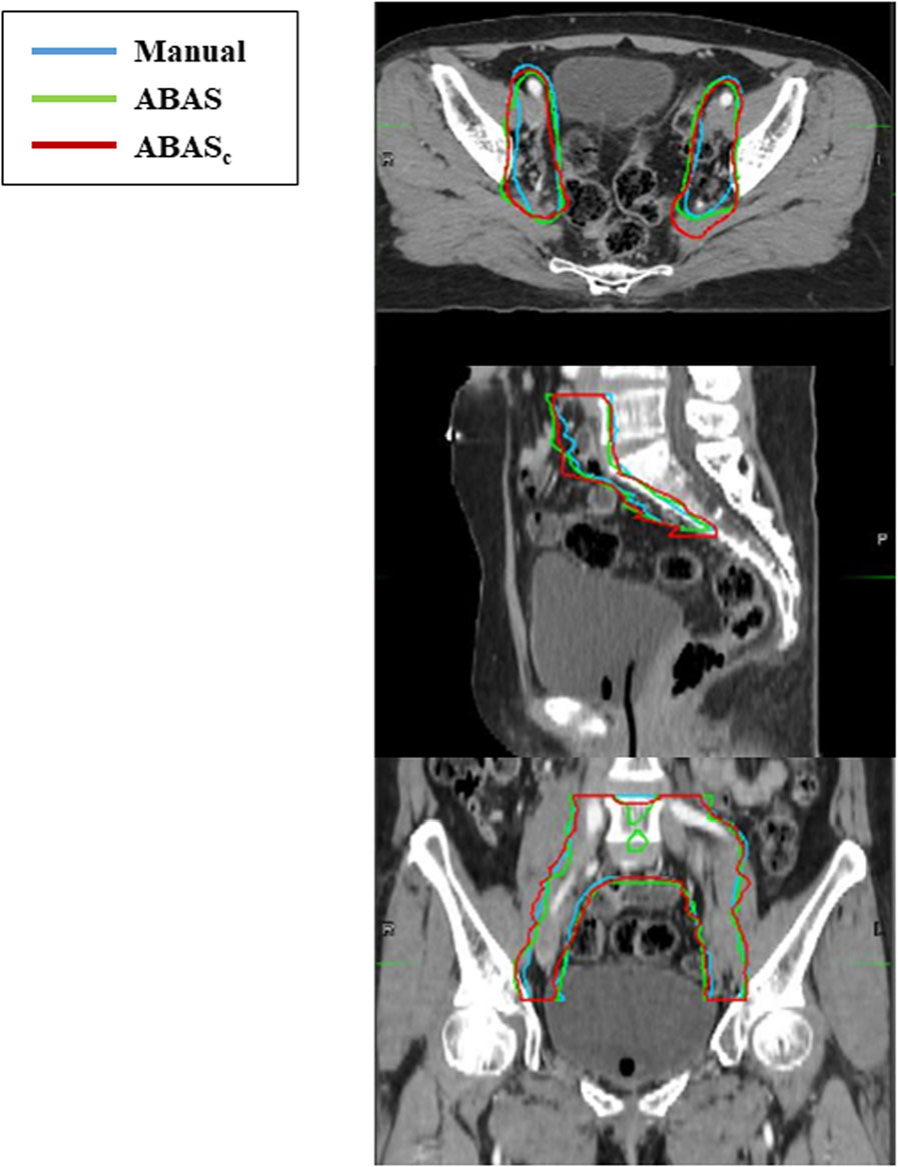
Auto-segmented contour results for clinical target volume. Atlas-based auto-segmentation alone (ABAS) and manual correction after ABAS (ABAS_c_)

**Fig. 2:**
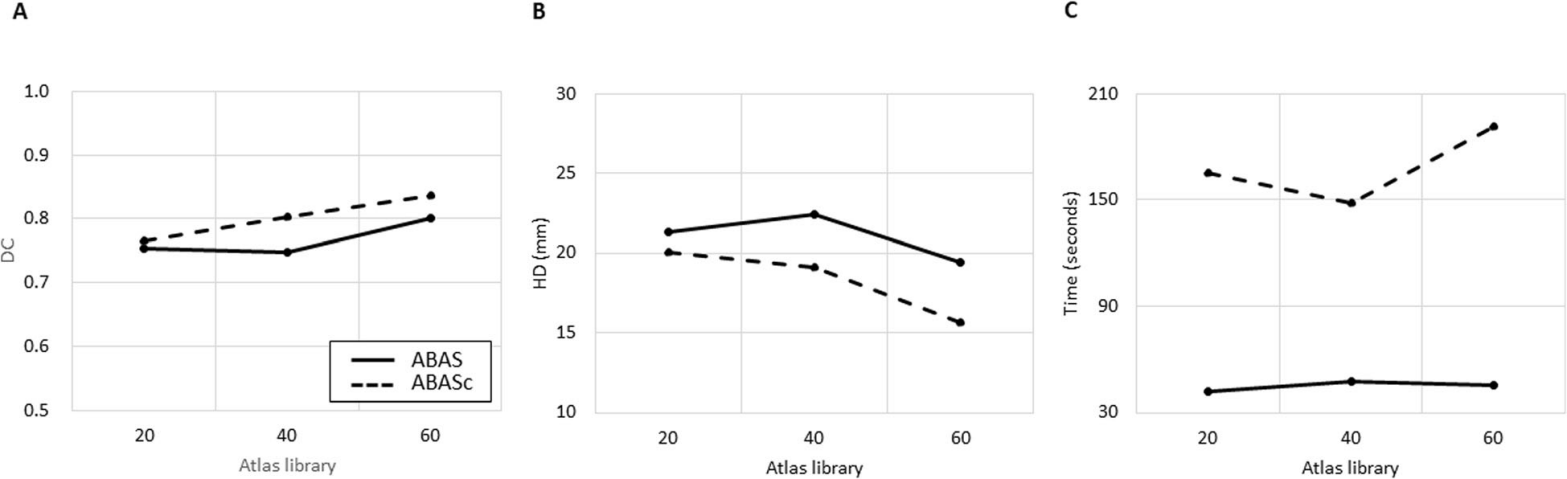
Comparison of metrics among atlas libraries for clinical target volume. Atlas-based auto-segmentation alone (ABAS) is represented as a bold line and manual correction after ABAS (ABAS_c_) is represented as a dashed line. **a** Mean Dice’s coefficient (DC) for target volume. **b** Mean Hausdorff distance (HD) for target volume. **c** Mean operation time for target volume

The atlas library with 60 sets produced the best results for ABAS-CTV, with a mean DC of 0.79 and a mean HD of 19.7 mm. The results were consistent with those obtained by ABAS_c_, with the mean DC ranging from 0.82–0.84 and HD ranging from 15.6–17.4 mm (Fig. 1). Mean DC and HD values for ABAS-CTV and ABAS_c_-CTV are summarized in Table 1. The performance of ABAS_c_-CTV was better than that of ABAS-CTV, regardless of the library size, based on DC and HD (all *p* < 0.017, Fig. 2a, b). For both ABAS-CTV and ABAS_c_-CTV, there was a trend of a higher degree of agreement with an increasing number of sets in each library (Fig. 2a-b).

**Table 1:** Mean Dice’s coefficient and Hausdorff distance values for multiple atlas libraries of clinical target volumes

	Size of atlas library		
	20	40	60
ABAS-CTV			
DC (95% CI)	0.75 (0.73–0.77)	0.75 (0.72–0.78)	0.79 (0.77–0.80)
*p*-value	ref.	0.656	0.012 (0.002^++^)
HD (95% CI)	21.3 (18.8–24.0)	23.8 (20.9–26.9)	19.7 (17.8–22.0)
*p*-value	ref.	0.137	0.002 (0.012^++^)
ABAS_c_-CTV			
DC (95% CI)	0.82 (0.80–0.82)	0.83 (0.81–0.84)	0.84 (0.82–0.85)
*p*-value	ref.	0.555	0.010 (0.015^++^)
*p*-value*	0.001	0.009	0.004
HD (95% CI)	17.4 (15.1–20.1)	17.1 (15.0–19.1)	15.6 (14.2–17.1)
*p*-value	ref.	0.046	0.200 (0.001^++^)
*p*-value*	0.005	0.002	0.014

*Abbreviations*: *ABAS* atlas-based auto-segmentation alone, *ABAS*_*c*_ manually corrected ABAS, *CTV* clinical target volume, *DC* Dice’s coefficient, *HD* Hausdorff distance, *CI* confidence interval

* Comparison between ABAS and ABAS_c_

^++^ Comparison between atlas size 40 and 60

**Fig. 3:**
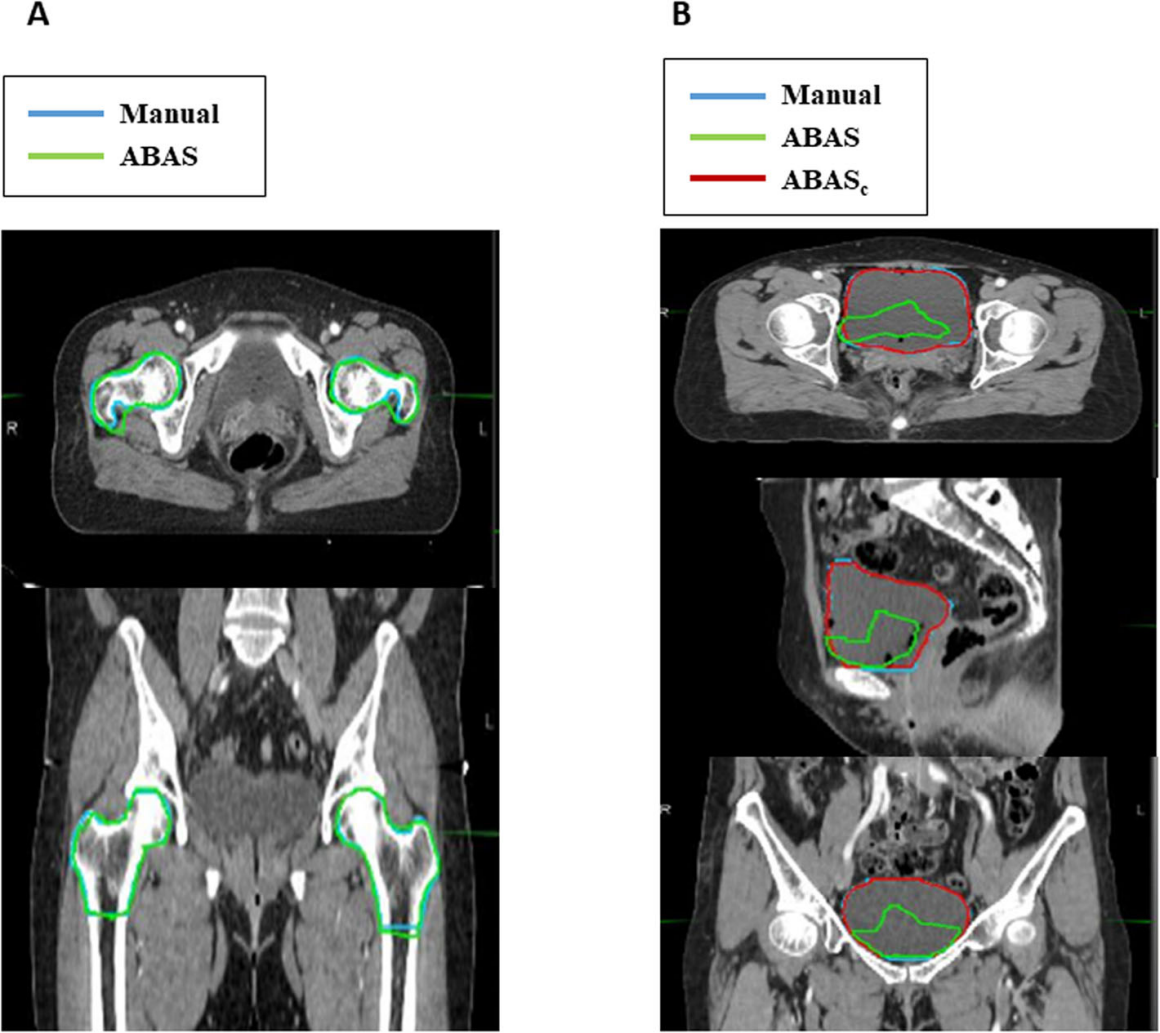
Auto-segmented contour results in organs-at-risk. **a** Auto-segmented contour results in the femur. **b** Auto-segmented contour results in the bladder

**Fig. 4:**
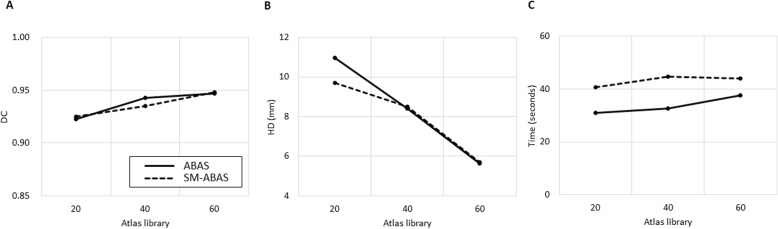
Comparison of metrics among atlas libraries for the femur. Atlas-based auto-segmentation alone (ABAS) is represented as a bold line and simultaneous ABAS for bilateral femurs (SM-ABAS) is represented as a dashed line. **a** Mean Dice’s coefficient (DC), **b** mean Hausdorff distance (HD), and **c** mean time of operation

**Fig. 5:**
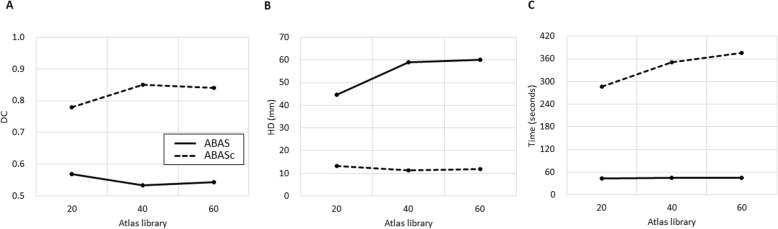
Comparison of metrics among atlas libraries for the bladder. Atlas-based auto-segmentation alone (ABAS) is represented as a bold line and manual correction after ABAS (ABAS_c_) is represented as a dashed line. **a** Mean Dice’s coefficient (DC), **b** mean Hausdorff distance (HD), and **c** mean time of operation

ABAS-Femur (Fig. 3a) showed a high degree of agreement, with a mean DC > 0.90 and HD < 10 mm in all atlas libraries (Fig. 4a, b). Mean DC and HD for the femur and bladder are summarized in Table 2. There was no significant improvement in accuracy according to the size of the atlas library. The results of simultaneous ABAS-Bilateral-femurs also demonstrated a good agreement (mean DC ranging from 0.93 to 0.95 and HD ranging from 5.7 to 9.7 mm) and did not have reduced accuracy when compared to one-sided ABAS-Femur (all *p* > 0.017). The performance of ABAS-Bladder (Fig. 3b) showed the lowest agreement, with a mean DC < 0.6 and a mean HD > 40 mm, in all atlas libraries (Fig. 5a, b). For the bladder, significant improvement after manual correction was found in both DC (mean ranging from 0.78 to 0.85) and HD (mean ranging from 11.3 to 13.2 mm).

**Table 2:** Mean Dice’s coefficient and Hausdorff distance values for multiple atlas libraries of normal organs

	Size of atlas library		
	20	40	60
Femur			
DC (95% CI)	0.92 (0.90–0.95)	0.94 (0.93–0.96)	0.95 (0.94–0.96)
*p*-value	ref.	0.289	0.335 (0.335^++^)
HD (95% CI)	10.0 (7.8–15.4)	8.4 (6.0–10.8)	5.6 (4.0–7.4)
*p*-value	ref.	0.285	0.046 (0.024^++^)
Femur-sm			
DC (95% CI)	0.93 (0.91–0.94)	0.94 (0.93–0.95)	0.95 (0.93–0.96)
*p*-value	ref.	0.298	0.304 (0.139^++^)
*p*-value*	0.341	0.798	0.782
HD (95% CI)	9.7 (7.2–12.2)	8.5 (6.3–10.8)	5.7 (4.5–7.1)
*p*-value	ref.	0.466	0.023 (0.013^++^)
*p*-value*	0.162	0.869	0.794
ABAS-Bladder			
DC (95% CI)	0.57 (0.47–0.65)	0.53 (0.45–0.62)	0.54 (0.44–0.63)
*p*-value	ref.	0.454	0.803 (0.096^++^)
HD (95% CI)	44.6 (31.0–66.7)	59.1 (39.1–82.1)	60.2 (40.2–84.0)
*p*-value	ref.	0.121	0.883 (0.024^++^)
ABAS_c_-Bladder			
DC (95% CI)	0.78 (0.73–0.83)	0.85 (0.81–0.88)	0.84 (0.82–0.87)
*p*-value	ref.	0.001	0.006 (0.398^++^)
*p*-value**	0.051	< 0.001	0.001
HD (95% CI)	13.2 (11.8–14.6)	11.3 (10.6–12.0)	11.9 (11.1–12.9)
*p*-value	ref.	0.031	0.085 (0.282^++^)
*p*-value**	0.196	0.020	0.007

*Abbreviations ABAS* atlas-based auto-segmentation alone, *ABAS*_*c*_ manually corrected ABAS, *sm* simultaneous, *DC* Dice’s coefficient, *HD* Hausdorff distance, *CI* confidence interval

* Comparison between Femur and Femur-sm

** Comparison between ABAS and ABAS_c_

^++^Comparison between atlas sets sized 40 and 60

### Time

The shortest mean time was achieved by ABAS (*n* = 20) with a mean of 41.6 (95% CI 40.4–42.6) seconds for CTV **(**Fig. 2c), 31.1 (95% CI 28.4–34.4) seconds for the femur (Fig. 4c), and 42.8 (95% CI 41.1–44.6) seconds for the bladder (Fig. 5c); these time values were significantly lower compared to those of M-CTV (749.4, 95% CI 622.3–860.4 s), M-Femur (128.1, 95% CI 122.3–135.1 s), and M-Bladder (142.0, 95% CI 115.2–171.4 s, all *p* < 0.001). The mean time values spent on each process for CTV, femur, and bladder are summarized in Table 3. Although the mean time needed for ABAS for all targets increased as the number of sets in each library increased (Fig. 2c), there was still a significant time reduction compared with that in manual contouring (all *p* < 0.001). Conversely, ABAS_c_-CTV showed no statistical difference in the time spent according to the number of sets in the library. Although there was a significant difference in the time spent between ABAS-CTV and ABAS_c_-CTV, the time spent in ABAS_c_ was still significantly shorter than that spent in M-CTV. Although simultaneous ABAS-Bilateral-femurs was associated with a significantly longer time than ABAS-Femur, significant time reduction was achieved compared with that in M-Femur. Additionally, the processing time for ABAS-Bilateral-femurs was less than double the time required to process ABAS-Femur. In contrast to ABAS_c_-CTV and simultaneous ABAS-Bilateral-femurs, ABAS_c_-Bladder had a significantly prolonged process time compared with that of M-Bladder (*p* < 0.001).

**Table 3:** Mean time values for multiple atlas libraries of clinical target volumes and normal organs

	Size of atlas library			Manual contouring
	20	40	60	
ABAS-CTV				
Time (95% CI)	41.6 (40.4–42.6)	47.6 (46.0–49.2)	45.1 (44.0–46.1)	749.4 (622.3–860.4)
*p*-value	ref.	0.001	0.013 (0.023^++^)	
ABAS_c_-CTV				
Time (95% CI)	164.8 (139.3–195.2)	148.1 (131.9–164.0)	191.1 (162.8–221.1)	749.4 (622.3–860.4)
*p*-value	ref.	0.220	0.187 (0.038^++^)	
*p*-value*	0.001	0.001	0.001	
Femur				
Time (95% CI)	31.1 (28.4–34.4)	32.7 (31.3–34.1)	37.5 (36.3–38.8)	128.1 (122.3–135.1)
*p*-value	ref.	0.316	0.005 (0.003^++^)	
Femur-sm				
Time (95% CI)	60.7 (55.9–65.4)	64.8 (63.3–66.2)	74.1 (72.4–75.9)	256.0 (244.5–262.1)
*p*-value	ref.	0.125	0.002 (0.001^++^)	
*p*-value**	0.001	0.001	0.001	
ABAS-bladder				
Time (95% CI)	42.8 (41.1–44.6)	44.6 (43.3–45.9)	45.6 (43.9–47.2)	142.0 (115.2–171.4)
*p*-value	ref.	0.052	0.010 (0.108^++^)	
ABAS_c_-bladder				
Time (95% CI)	286.3 (256.3–315.6)	350.7 (319.3–382.5)	375.7 (345.5–405.0)	142.0 (115.2–171.4)
*p*-value	ref.	0.001	0.001 (0.001^++^)	
*p*-value*	0.001	0.001	0.001	

*Abbreviations*: *ABAS* atlas-based auto-segmentation alone, *ABASc* manually corrected ABAS, *CTV* clinical target volume, *DC* Dice’s coefficient, *HD* Hausdorff distance, *CI* confidence interval

* Comparison between ABAS and ABAS_c_

** Comparison between Femur and Femur-sm

++ Comparison between atlas size 40 and 60

## Discussion

In this study, we investigated the feasibility of atlas-based auto-contouring in the delineation of target volume and OAR for adjuvant RT in gynecologic cancers. We evaluated the performance of ABAS using DC and HD. The mean DC and HD values of ABAS_c_-CTV were improved compared with those of ABAS-CTV, but with prolonged process time. Both ABAS-Femur and ABAS-Bilateral-femurs exhibited accurate delineation (DC > 0.90, HD ≤10.0 mm) with reduced time compared with that achieved with M-Femur. However, ABAS-Bladder performed poorly with a DC of 0.54, and ABAS_c_-Bladder took more time than M-Bladder.

Delineation of target volume and OAR is the only procedure that still entirely depends on manual process in RT planning and is a time-consuming step. Apart from the time consumption issue, it has been reported that manual contouring has its limitation due to inter- and intra-observer variability [5, 6]. Although ABAS has been introduced and investigated previously, earlier phases of the technology did not have satisfactory accuracy [12]. However, recent studies have shown the feasibility of ABAS in patients with head and neck cancers [7, 13, 27–32], prostate cancer [12–17], endometrial cancer [18], rectal cancer [33, 34], and breast cancer [16, 33, 35]. Wong et al. [15] also reported that multiple-ABAS had better accuracy than single-ABAS, and they demonstrated that the single-atlas approach was sensitive to the library size. Consistent with previous reports, we showed that the segmentation accuracy of ABAS-CTV improved with increasing library size in the multiple-atlas approach. However, manual editing on the basis of ABAS demonstrated better accuracy than ABAS alone and reduced the time spent compared with that spent on manual contouring. In other words, ABAS could assist physicians in delineating the target and OAR accurately and effectively rather than surpassing manual contouring. Recently, the scope of auto-segmentation has been expanded to artificial intelligence (AI)-based contouring using deep learning algorithms. The aid of AI tools beyond ABAS had a positive impact on contouring accuracy. Lin et al. [36] demonstrated that physicians could reduce the time spent by nearly 40% (from 30.2 min to 18.3 min) and intra-observer variation by nearly 36% with AI assistance. Lately, there was a growing evidence [37–39] that the convergence of deep learning algorithms and manual work by clinicians could improve accuracy, productivity, and efficiency in the practice of medicine [40].

The segmentation accuracy between the femur and bladder differed significantly in ABAS. The suboptimal results of ABAS-Bladder were consistent with those shown in previous reports [34]. Because ABAS supported by MIM software includes template alignment and best matching contour searching processes using deformable image registration, organs isodense with their surroundings are not suitable subjects for ABAS. Due to its inferior segmentation accuracy at baseline, ABAS_c_-Bladder required even more time to achieve results comparable to those of M-Bladder. To overcome this limitation of ABAS-Bladder, detection of a contrast agent in the bladder could contribute to a more robust ABAS-Bladder due to strong gradients in gray levels [17]. Additionally, further investigations using advanced techniques for auto-contouring, like deep learning algorithms, are needed for precise contouring of isodense OARs, such as the bladder or bowels.

Along with the accuracy of ABAS-Femur, the results of simultaneous ABAS-Bilateral-femurs were satisfactory in terms of both accuracy and efficiency. Unlike ABAS-CTV, the difference in the performance of both ABAS-Femur and ABAS-Bilateral-femurs did not vary drastically with increasing library size and may not be improved by even larger library sizes. In contrast with the bladder, the significant difference in the bone density compared with that of the surrounding soft tissues could make femurs suitable subjects for ABAS [16]. We suggest that the size of the atlas library is not an independent factor in determining the quality of auto-segmentation; the quality could also be attributed to the density contrast. Furthermore, it does not seem necessary to construct separate libraries according to the laterality of some OARs with distinct contrast, such as the femurs and mandible.

There are several limitations in the current study. First, there was some selection bias in terms of the CT samples, despite the random selection of the 75 samples. Furthermore, statistical analysis with a small cohort has its limitation in terms of overfitting of the data (type II error). Therefore, further investigations including a large number of independent CT sets are needed for evaluating the efficacy of the currently built ABAS library. However, we demonstrated both the potential benefit of ABAS combined with manual modification and the disparity in the accuracy of ABAS according to the soft tissue density. Herein, the results of 15 patients in the test set could support the hypothesis that manual correction is necessary even in the well-known ABAS algorithm, and differences in soft-tissue density should be considered in the implementation of ABAS. Second, ABAS is limited by its inflexibility, as segmentation is limited to the specific shapes defined by the statistical model [10]. Although we evaluated accuracy based on DC, the DC value could overestimate the accuracy; Tsuji et al. [29] found that a sensitivity index, rather than DC, could be an informative predictive factor. Voet et al. [41] demonstrated that planning based on ABAS was suboptimal, exhibiting suboptimal dose coverage of up to 11 Gy, despite a high DC of 0.8. Conversely, ABAS could be valuable in clinical application if the DC of ABAS_c_-CTV is less than 0.8. In addition, a further investigation using data from different institutions is crucial to validate this approach in real clinical practice. However, it has been proposed that the accuracy of segmentation highly relies on the training set, and amendment of data from other institutions can improve the performance of segmentation [42]. Although recent advances in auto-segmentation have entered the fourth generation with deep learning algorithms [43], ABAS, which is the 3rd generation of auto-segmentation, could be easily utilized even in an institution with limited resources.

## Conclusions

Based on this evaluation of the ABAS algorithm with individual institutional data, we recommend ABAS combined with manual corrections for CTV in clinical use for postoperative RT for gynecologic cancers. ABAS of the bilateral femurs could be considered for clinical use without manual correction. Highly variable, structures that are isodense with the surrounding tissue, such as the bladder, should be contoured manually rather than with ABAS. The implementation of ABAS with manual adjustment in daily clinical practice could change the workflow of physicians even in institutions with limited resources; however, further implementation of and investigations into AI with deep learning algorithms are still needed to improve the accuracy and efficiency of auto-contouring. In addition, further investigations on the feasibility of RT plans based on ABAS-generated contours for both CTV and OAR are still needed.

## Supplementary information

**Additional file 1.**

**Additional file 2.**

## Data Availability

Data availability is limited due to institutional data protection law and confidentiality of patient data.

## Abbreviations

ABAS: Atlas-based auto-segmentation
ABASc: Manually corrected atlas-based auto-segmentation
AI: Artificial intelligence
CTV: Clinical target volume
DC: Dice’s coefficient
HD: Hausdorff distance
IMRT: Intensity-modulated radiotherapy
M-CTV: Manually contoured clinical target volume
OAR: Organ(s)-at-risk
RT: Radiotherapy
