# Supplementary material for: Atlas-based auto-segmentation for postoperative radiotherapy planning in endometrial and cervical cancers
Source: Radiat Oncol. 2020 May 13;15:106. doi: 10.1186/s13014-020-01562-y (PMC7218589; doi:10.1186/s13014-020-01562-y)
Supplement: Supplementary file 1 — Additional file 1. [file 13014_2020_1562_MOESM1_ESM.docx]

**Additional file 1.** Study scheme


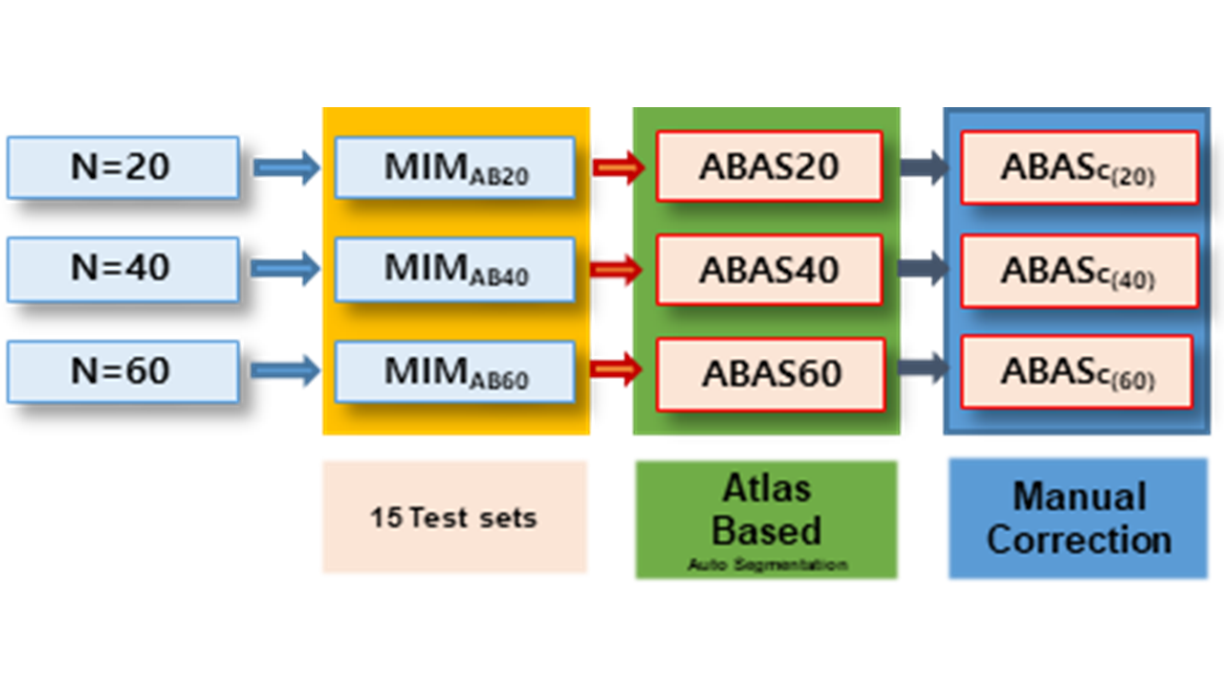


Abbreviations: ABAS, atlas-based auto-segmentation alone; ABASc, manually corrected ABAS.
