# Supplementary material for: Atlas-based auto-segmentation for postoperative radiotherapy planning in endometrial and cervical cancers
Source: Radiat Oncol. 2020 May 13;15:106. doi: 10.1186/s13014-020-01562-y (PMC7218589; doi:10.1186/s13014-020-01562-y)
Supplement: Supplementary file 2 — Additional file 2. [file 13014_2020_1562_MOESM2_ESM.docx]

**Additional file 2.** Baseline characteristics*

|  |  | Test  N=15 | Size of atlas library | | |
| --- | --- | --- | --- | --- | --- |
|  |  |  | N = 20 | N = 40 | N = 60 |
| Mean age (SD) |  | 54.2 (8.8) | 51.1 (11.3) | 54.4 (10.7) | 52.8 (10.8) |
| Primary, n (%) | Uterine cervix | 7 (46.7) | 9 (45.0) | 19 (47.5) | 28 (46.7) |
|  | Endometrium | 8 (53.3) | 11 (55.0) | 21 (52.5) | 32 (53.3) |
| Mean height (SD), cm |  | 158.0 (6.4) | 158.0 (5.9) | 156.7 (5.4) | 157.4 (5.9) |
| Mean weight (SD), kg |  | 61.7 (9.7) | 61.5 (8.0) | 60.3 (7.4) | 60.8 (8.2) |
| Mean BMI (SD), kg/m2 |  | 24.7 (3.6) | 24.6 (3.0) | 24.6 (2.8) | 24.5 (3.0) |

*All *p*-values > 0.05

**Abbreviations:** BMI, body mass index; SD, standard deviations
